# Magnitude and Its Associated Factors of Urinary Tract Infection among Adult Patients Attending Tigray Region Hospitals, Northern Ethiopia, 2019

**DOI:** 10.1155/2020/8896990

**Published:** 2020-07-28

**Authors:** Abrha Hailay, Kidane Zereabruk, Guesh Mebrahtom, Woldu Aberhe, Degena Bahrey

**Affiliations:** School of Nursing, Aksum University, Aksum, Ethiopia

## Abstract

**Background:**

Urinary tract infection is a major public health problem in terms of morbidity and mortality worldwide. It ranks as the number one infection which leads to an antibiotic prescription after a physician's visit. However, there are limited studies done on UTI in Ethiopia. Hence, this study was aimed to assess the magnitude of urinary tract infection and its associated factors among adult patients attending hospitals of the Tigray region, Ethiopia. *Methods and Material*. A hospital-based cross-sectional study was conducted from April to May 2019. Systematic random sampling technique was used to select 472 participants from five randomly selected hospitals in Tigray region. A pretested structured questionnaire through face-to-face interview and patient chart review checklist was used to collect data. Data were analyzed by SPSS version 21. A binary logistic regression model was used to test the association between dependent and independent variables.

**Result:**

The magnitude of urinary tract infection was 86 (18.2%) (95% CI: 14.6%–21.6%). After adjustment of the independent variables, the significant factors associated with urinary tract infection were being female (AOR = 3.50; 95% CI: 1.88–6.51), urine passing frequency < five times in a day (AOR = 2.32; 95% CI: 1.08–4.96), having diabetes mellitus (AOR = 4.03; 95% CI: 1.69–9.63), history of urinary tract infection (AOR = 4.40; 95% CI: 2.31–8.39), <7 glasses of water intake per day (AOR = 2.16; 95% CI: 1.02–4.58), and history of urinary obstructive diseases (AOR = 2.67; 95% CI: 1.03–6.90). *Conclusion and Recommendation*. The magnitude of urinary tract infection was considerably high. The factors associated with urinary tract infection were sex, less urine passing frequency, diabetes mellitus, low water intake, history of urinary tract infection, and urinary obstructive diseases. Therefore, patients having DM, previous history of UTI, and urinary obstructive diseases should be routinely screened for urinary tract infection and provided with education on voiding urine at least five times a day and on increasing daily water intake.

## 1. Introduction

Urinary tract infection (UTI) is the presence of microorganisms (predominantly bacteria) in urine along with urinary symptoms of dysuria, frequency, urgency, and occasionally suprapubic tenderness [1]. Urinary tract infection (UTI) is among the most common bacterial infections acquired in the community and in hospitals. It remains a major public health problem in terms of morbidity with an estimated 150 million cases annually worldwide with global costing greater than 6 billion US dollars [2]. Although this infection affects both genders, women are the most vulnerable maybe due to their anatomy and reproductive physiology [3].

UTI is a common disease in developing countries with an estimated incidence of at least 8.3 million doctor visits yearly [4]. Besides, UTI ranks as the number one infection that leads to an antibiotic prescription after a physician's visit [5]. Recurrence of UTI is also most common, with nearly half of people getting a second infection within a year [6].

Risk factors associated with developing UTI include age-related changes to the genitourinary tract, comorbid conditions, and instrumentation required to manage bladder voiding [7]. The prevalence also increases with advancing age, catheterization, sexual activity, menopause, and urinary obstruction problems [3].

UTI will have a high contribution to the emerging of antibiotic resistance and other health complications. However, there are limited studies done on UTI in Ethiopia since the previous studies were done among susceptible groups, i.e., DM patients and pregnant mothers. This study was done in all patents attending OPD including DM and pregnant women. Therefore, this study was aimed to assess the magnitude of UTI and its associated factors among adult patients attending in Tigray region hospitals, Ethiopia.

This study also may help as the literature for decision-makers and program planners at the time to design and implementation of intervention. Educational centers, health institutions, and health care providers will gain a better insight into the magnitude and factors associated with UTI. Moreover, this study could be used as a baseline for future researchers and be a cue for further studies to be done on UTI.

The research questions for this study were as follows:

1. What is the magnitude of urinary tract infection cases among OPD-attending patients in Tigray region hospitals?
2. What are the potential risk factors of urinary tract infection among OPD-attending patients in Tigray region hospitals?

## 2. Methods and Material

### 2.1. Study Area, Period, and Design

A hospital-based cross-sectional study design was conducted in Tigray region hospitals from April to May 2019. Tigray region is found in the northern part of Ethiopia. Tigray's surface area is 53,638 km^2^, and its current population size according to the 2007 Population and Housing Census projection is 6.8 million (3.2 million male and 3.6 million female). The highlands (11.5% däga and 40.5% wäyna däga) have the highest population density and the much less densely populated in lowlands of Tigray (qwälla) comprise 48% of Tigray population.

Tigray region has 2 referral hospitals and 15 general hospitals; of those hospitals, five hospitals were selected using simple random sampling for this study (Ayder Comprehensive Specialized Hospital, Aksum Referral Hospital, Adigrat General Hospital, Lemlem-Carl General Hospital, and Shule General Hospital).

### 2.2. Source Population

The source population comprised of all adult patients who were attending the OPD of Tigray region hospitals during the data collection period.

### 2.3. Study Population

The study population comprised of all selected adult patients who were attending OPD of the selected hospitals in the Tigray region during the data collection period.

### 2.4. Eligibility Criteria

All adult patients not on any antibiotics therapy and willing to participate were included in the study while critically ill patients and those on any antibiotic therapy were excluded from the study.

### 2.5. Sample Size Determination

The required sample size was determined by using single and double population proportions. Finally, the single proportion formula was used to determine the sample size since it gave the maximum sample size. By considering 95% confidence interval, prevalence was 16.7% according to Metu Karl Heinz Referral Hospital, Southwest Ethiopia [8], and the margin of error was 5%. Finally, the estimated sample size was 214 plus 10% nonresponse rate, that is, 236 by considering the design effect of 2, and the total sample size was 472.

### 2.6. Sampling Technique and Procedure

First five hospitals were selected using simple random sampling technique from the eligible hospitals. The study participants were selected using a systematic random sampling method from the patients who were attending the OPD during the data collection period. The previous year's average monthly patient flow to OPDs of the selected five hospitals was collected. A simple random sampling (lottery) method was used to select the first number, and thereafter, patients were picked at a regular interval (sample interval) to meet the required sample size. The sample interval (SI) was determined by dividing the average number of monthly patients flow (N) to each OPD of the selected hospital by the proportional sample size (*n*) given to the selected hospital (*K* = *N*/*n*).

### 2.7. Data Collection Tools and Instruments

A structured questionnaire and checklist were used to collect data. An interviewer-administered questionnaire was used to collect primary data from study participants. Moreover, the chart review checklist was also used to collect secondary data from patient cards. The weight and height of the study participant were measured using a digital weight scale and stadiometer, respectively, to calculate the body mass index (BMI).

### 2.8. Data Collection Procedure

Ten BSc nurses as data collectors and five senior BSc nurses as supervisors were recruited. Data were collected by face-to-face interviews and reviewing of patient medical cards. Participants were interviewed after getting the medical service. The weight and height were measured with participants standing without shoes and wearing light clothing. Participants were standing upright with the head in the Frankfort plane for height measurement. Bodyweight (kg) was measured using an electronic scale to the nearest 10 g, and the scale was calibrated to zero before weight measurement. Standing height was measured using a wall stadiometer to the nearest 0.1 cm. The BMI was calculated as body weight (kg)/height (m^2^). Finally, the diagnosis of UTI was confirmed by checking the medical card of the patent.

### 2.9. Study Variables

#### 2.9.1. Dependent Variable

The dependent variable was urinary tract infection.

#### 2.9.2. Independent Variables

The independent variables included sociodemographic characteristics (sex, age, occupation, residence, level of education, marital status, and weather condition).

Behavioral and dietary factors were as follows: daily water intake, Coca-Cola, tea and/or coffee intake, circumcision, urination before and after coitus, washing before and after coitus, high dietary salt intake, cigarette smoking, urine passing frequency, and alcohol intake.

Health-related factors were as follows: BMI status, DM, HTN, catheterization, previous history of UTI, SCI, and urinary obstructive disease urolithiasis.

### 2.10. Operational Definition

  Urinary Tract Infections. In this study, the magnitude of urinary tract infection and its associated factors were assessed in patients who had confirmed the presence of infection in any part of the urinary tract (renal calyx or pelvis, ureter, bladder, prostate, and/or urethra) by a physician based on the information obtained from history, “physical examination,” laboratory investigation, and/or radiographic studies [1].
  BMI Status. Weight status was classified into four categories: underweight (BMI < 18.5), normal weight (BMI: 18.5–24.9 kg/m^2^), overweight (BMI: 25–29.9 kg/m^2^), and obese (BMI ≥ 30 kg/m^2^) [9].
  Urinary Obstructive Disease. In this study, urinary obstructive diseases included benign prostatic hyperplasia/cancer, urethral stricture, and trauma to urinary tract [10].

### 2.11. Data Quality Assurance

Data quality was ensured by providing training to data collectors and supervisors about the research objective, participant selection method, eligible study subjects, data collection tools, and procedures. The data collection tools were first developed in English then translated into Tigrigna language, finally translated back into English to check its consistency in the meaning of words by a language expert. A pretest was done on five percent of the total sample size at the Mekelle General Hospital one month before the actual data collection period. Based on the result of the pretest, necessary corrections and amendments were undertaken on the data collection tools.

### 2.12. Data Processing and Analysis

The data were coded and entered into EpiData Manager version 4.4.3.1 and then exported to SPSS version 21 for analysis. The data were cleaned using frequencies and cross-tabulation before regression analysis. Descriptive statistics was computed, and the result was summarized by texts, tables, frequency, percentages, and charts and presented for continuous and discrete data in terms of mean and standard deviation for those normally distributed data.

The Hosmer–Lemeshow test was used to check the fitness model, and it was fitted (*P* = 0.35). The binary logistic regression model was used to test the association between dependent and independent variables. All variables with *P* value <0.25 in the binary regression analyses were included in the multivariable regression analysis. The degree of the association was interpreted by using the adjusted odds ratio with 95% confidence intervals, and the significance level was declared at *P* value <0.05. Multicollinearity was checked using a variance inflation factor (VIF<1.89) and tolerance test (>0.5).

## 3. Results

### 3.1. Sociodemographic Characteristics

A total of 472 participants were included in this study with a response rate of 100%. This study consisted of 261(55.3%) males and 256 (56.1%) married respondents. 251(53.2%) of the respondents were living in urban areas, and 392 respondents (83.1%) were using living in lowland weather conditions (Table 1). The mean and standard deviation age of respondents was 42 ± 16 years with minimum of 18 years and a maximum of 89 years old.

### 3.2. Health-Related Factors

Respondents were assessed for the history of different medical history. Based on the assessment, 43 (9.1%), 45 (9.5%), and 70 (14.8%) of the respondents had overweight, DM, and hypertension, respectively. Nine (4.3%) of female respondents and 8 (3.1%) of male respondents were pregnant and not circumcised (Table 2).

### 3.3. Behavioral and Dietary Factors

Four hundred eleven (87.1%) and 217 (46%) did not urinate before and after sexual intercourse. Regarding washing after and/or before sexual intercourse, 234 (49.1%) and 236 (50%) did not practice washing before and after sexual intercourse, respectively. Four hundred three (85.4%) respondents consumed Coca-Cola, tea, and/or coffee. About 73% of respondents passed urine less than five times per day, and 335 (71%) of study participants consumed <7 glasses of water per day (Table 3). The mean and standard deviation of urine passing frequency per day was 4 ± 1 times a day.

### 3.4. Prevalence of UTI

**Figure 1:**
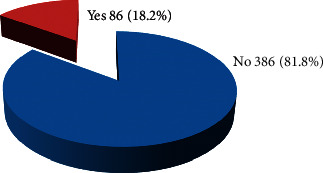
Magnitude of UTI among adult patients attending the OPD in Tigray region hospitals, Ethiopia, 2019.

The overall prevalence of UTI among patients attending in Tigray hospitals was 86 (18.2%) (95% CI: 14.6%–21.6%) (Figure 1). Of these, 58 (67.4%) were females and 28 (32.6%) were males.

### 3.5. Factors Associated with UTI among Outpatient Department-Attending Patients

In bivariate regression analysis, being female; rural residence; lowland weather condition; less daily water intake; Coca-Cola, tea, and/or coffee intake; urine passing frequency < five times a day; high dietary salt intake; having DM, hypertension, and spinal cord injury; history of catheterization; history of UTI; history of urinary obstructive disease; and history of urolithiasis were statistically associated with UTI at a *P* value of less than 0.25.

In multivariable regression analysis, being female, urine passing frequency < five times a day, less daily water intake, having DM and urinary obstructive disease, and history of previous UTI were statistically associated with UTI at a *P* value of less than 0.05, but the other variables were not found statistically significant.

Female respondents were found 3.5 (AOR = 3.5; 95% CI: 1.88–6.51) times more likely to have UTI compared to their male counterparts. The odds of having UTI were 2.3 (AOR = 2.32; 95% CI: 1.08–4.96) times more among respondents who had passed urine < five times in a day compared to their counterpart. Respondents who had the urinary obstructive disease were 2.7 (AOR = 2.67; 95% CI: 1.03–6.9) times more likely to develop UTI than those who do not have a history of urinary obstructive disease. The odds of having UTI were 4 (AOR = 4.03; 95% CI: 1.69–9.63) times higher among respondents who have DM than participants without DM. The odds of having UTI were 2 (AOR = 2.16; 95% CI: 1.02–4.58) times higher among respondents who drunk ≤ 7 glasses of water than participants who drunk >7 glasses of water. Besides, the odds of having UTI were 4.4 (AOR = 4.40; 95% CI: 2.31–8.39) times more among those who have a history of UTI patients compared to those who did not have a history of UTI (Table 4).

## 4. Discussion

In this study, the prevalence of UTI among adult patients attending the OPD was 18.2% and the factors associated with urinary tract infection were being female, less urine passing frequency, diabetes mellitus, less water intake per day, history of urinary tract infection, and urinary obstructive diseases.

The overall prevalence of UTI among OPD-attending patients was 86 (18.2%) (95% CI: 14.6%–21.6%). This finding is similar to the studies conducted in Ethiopia, Metu (16.7%), Gonder (17.8%), and Nekemte (16.5%) [8, 11, 12]. This finding is lower than the studies done in Arbaminch Hospital (Ethiopia) (33.8%), Egypt (29%), Nigeria (60%), India (63.5%), Saudi Arabia (25.3%), and Bangladesh (79.5%) [10, 13–16]. This difference could be due to the difference in the study population (the majority of the studies were done on the pregnant women and DM patients which are the most vulnerable groups) and study setting (done in both the inpatient and OPD), and they were done in a single institution (the referral hospital).

In this study, being female was significantly associated with UTI. This finding is supported by the studies done in Ethiopia (Metu and Arbaminch), Nigeria, India, Egypt, Saudi Arabia, and Bangladesh [8, 10, 13–17]. The anatomy of the female urogenital tract with the shorter urethra (1.5 inches compared to 8 inches in males) and proximity of urethral opening to the vagina and anus increases their risk for UTI [3]. Women have an increased susceptibility to vaginal colonization with uropathogens, which is due to a greater propensity for uropathogenic coliforms to adhere to uroepithelial cells [18].

Having DM was significantly associated with UTI in this study. This finding is similar to studies done in Ethiopia (Metu and Arbaminch), Egypt, Nigeria, India, Saudi Arabia, Bangladesh, and USA [5, 6, 8, 10, 13–16, 19]. This association could be due to the high blood sugar in patients having diabetes mellitus can cause high urine glucose content and defective host immune factors predispose to infection. Hyperglycemia causes neutrophil dysfunction by increasing intracellular calcium levels and interfering phagocytosis [20]. High blood sugar in the blood is also media for the growth of bacteria [9]. The increased risk of infection in diabetics can be partially explained by a decreased T-cell-mediated immune response and impaired neutrophil function among diabetics. Higher glucose concentrations in urine may also play a role in increased incidences of UTI in diabetics [21].

In this study, previous history of UTI was significantly associated with UTI. This finding is supported by the studies done in Metu, Ethiopia, Gonder, Ethiopia, Nekemte, Ethiopia, and Egypt [8, 11, 12, 15]. The possible explanation for the difference might be due to relapse of the infection as a result of ineffective treatment. UTI should be treated based on culture results and then use of prophylactic therapy. Unfortunately, symptoms of a UTI may not be typical, and other conditions can manifest similarly. Treatment of UTI with antibiotics is usually required, but there is an increasing awareness of the need for antimicrobial stewardship to avoid the misuse and overuse of antibiotics, even as patients are increasingly reluctant to take them [22]. Uropathogens of UTI are increasingly becoming resistant to currently available antibiotics [2].

In this study, the odds of developing UTI were significantly higher for patients who had drunk ≤  7 glasses of water than those who had drunk >7 glasses of water. This was consistent with previous studies conducted in Arbaminch, Ethiopia, and Egypt [10, 15]. This association could be increased intake of water can remove the microorganisms from the urinary tract in the form of urination [23]. Inadequate hydration can lead to serious complications ranging from UTI to chronic kidney diseases. A low fluid intake (<1 L/day) can lead to reduced physical and cognitive performances. Adequate hydration is obtained by balancing the water intake (80%) and endogenous water production (20%) with water losses [24].

In this study, urine passing frequency less than five times per day was significantly associated with UTI. This result is similar to the study done in India and Nigeria [6, 14]. This association could be because of less urine passing frequency can result in urine stasis (supersaturation of urine) and this will result in the accumulation of bacteria in the urinary system and result in returning of urine from the lower to upper urinary tract.

In this study, participants with a history of urinary obstructive diseases were significantly associated with the development of UTI. This finding is supported by the study done in Nigeria and Latin America [6, 19]. The possible explanation could be urinary obstructive diseases can result in urine voiding dysfunction, return back of urine to the upper urinary tract, and accumulation of urine in the urinary system that increases the accumulation of bacteria in the urine [25].

## 5. Conclusion and Recommendation

In this study, the prevalence of urinary tract infection was considerably high. The factors associated with UTI were sex, less urine passing frequency, DM, low water intake, history of urinary tract infection, and urinary obstructive diseases. Therefore, patients having DM, previous history of UTI, and urinary obstructive diseases should be routinely screened for urinary tract infection and provided with education on voiding urine at least five times a day and on increasing daily water intake. Recommendation for researchers is that it is better to study in the community at all to identify asymptomatic UTI cases using screening material.

## Figures and Tables

**Table 1:** Sociodemographic characteristics of adult patients attending the OPD in Tigray region hospitals, Ethiopia, 2019 (*N* = 472).

Characteristics		Frequency (*n*)	Percentage (%)
Sex	Male	261	55.3
	Female	211	44.7

Age	18-24 years	60	12.7
	25-34 years	119	25.2
	35-44 years	105	22.2
	45-54 years	75	15.9
	55-64 years	63	13.3
	>65 years	50	10.6

Education	No formal education	181	38.3
	Primary school (1–8)	86	18.3
	Secondary school (9–12)	87	18.4
	College and above	118	25.0

Marital status	Single	118	25.1
	Married	265	56.1
	Divorced	68	14.4
	Widowed	21	4.4

Occupation	Farmer	182	38.6
	Government employee	76	16.1
	Private employee	30	6.4
	Merchant	75	15.9
	Nonemployed	36	7.6
	Student	46	9.7
	Others^*∗*^	27	5.7

Residence	Rural	221	46.8
	Urban	251	53.2

Environmental weather	Highland	80	16.9
	Lowland	392	83.1

Others^*∗*^ = pensioner, housewife, and daily labors.

**Table 2:** Clinical characteristics among adult patients attending the OPD in Tigray region hospitals, Ethiopia, 2019 (*N* = 472).

Variables		Frequency (*n*)	Percentage (%)
BMI category	Underweight	31	6.6
	Normal (healthy) weight	398	84.3
	Overweight and obese	43	9.1

DM	No	427	90.5
	Yes	45	9.5

Hypertension	No	402	85.2
	yes	70	14.8

Spinal cord injury	No	395	83.7
	Yes	77	16.3

Previous history of UTI	No	389	82.4
	Yes	83	17.6

Previous history of urolithiasis	No	428	90.7
	Yes	44	9.3

Urinary obstructive disease	No	421	89.2
	Yes	51	10.8

History of catheterization	No	427	90.5
	Yes	45	9.5

If female, pregnancy *N* = 211	No	202	95.7
	Yes	9	4.3

If male, circumcision *N* = 261	No	8	3.1
	Yes	253	96.9

**Table 3:** Behavioral and dietary factors among adult patients attending the OPD in Tigray region hospitals, Ethiopia, 2019 (*N* = 472).

Variable		Frequency (*n*)	Percent (%)
Urinate before sexual intercourse	No	411	87.1
	Yes	61	12.9

Urinate after sexual intercourse	No	217	46.0
	Yes	254	54.0

Coca-Cola ,tea, and/or coffee	No	69	14.6
	Yes	403	85.4

Washing before sexual intercourse	No	234	49.6
	Yes	238	50.4

Washing after sexual intercourse	No	236	50.0
	Yes	236	50.0

Cigarette smoking	No	460	97.5
	Yes	12	2.5

Consume any standard alcohol and/or homebrewed alcohol	No	222	47.0
	Yes	250	53.0

Passing urine per day	<5 times per day	344	72.9
	≥5 times per day	128	27.1

Glass of water per day	<7 glasses of water	335	71.0
	>7 glasses of water	137	29.0

**Table 4:** Regression analysis of associated factors with urinary tract infection among adult patients attending the OPD in Tigray region hospitals, Ethiopia, 2019 (*N* = 472).

Variables	UTI		COR (95% CI)	AOR (95% CI)	*P* value
	No	Yes			
Sex					
Male	233	28	1.00	1.00	
Female	153	58	3.15 [1.92–5.17]	**3.5 [1.88–6.51]**	**<0.001**
Residence					
Urban	213	38	1.00	1.00	
Rural	173	48	1.55[0.97–2.49]	1.63 [0.91–2.88]	0.096
Weather condition					
Highland	75	5	1.00	1.00	
Lowland	311	81	3.90 [1.53–9.98]	2.72 [0.99–7.50]	0.052
Daily water intake					
>7 glasses of water	126	11	1.00	1.00	
≤7 glasses of water	260	75	3.30 [1.69–6.44]	**2.16 [1.02–4.58]**	**0.043**
Intake of Coca-Cola, tea, and coffee					
Yes	61	8	1.00	1.00	
No	325	78	1.83 [0.84–3.98]	1.60 [0.65–3.92]	0.306
Urine passing frequency					
≥5 times per day	118	10	1.00	1.00	
<5 times per day	268	76	3.35 [1.67–6.69]	**2.32 [1.08–4.96]**	**0.030**
History of urinary obstructive disease					
No	353	68	1.00	1.00	
Yes	33	18	2.83 [1.50–5.31]	**2.67 [1.03–6.90]**	**0.042**
Diabetes mellitus					
No	364	63	1.00	1.00	
Yes	22	23	6.04 [3.17–11.48]	**4.03 [1.69–9.63]**	**.002**
Hypertension					
No	338	64	1.00	1.00	
Yes	48	22	2.42 [1.36–4.28]	1.53 [0.68–3.43]	0.298
History of spinal cord injury					
No	343	46	1.00	1.00	
Yes	43	40	1.46 [0.81–2.64]	1.67 [0.71–3.96]	0.237
History of UTI					
No	343	46	1.00	1.00	
Yes	43	40	6.93 [4.08–11.77]	**4.40 [2.31–8.39]**	**<0.001**
History of catheterization					
No	357	70	1.00	1.00	
Yes	29	16	2.81 [1.45–5.45]	2.00 [0.86–4.67]	0.106
History of urolithiasis					
No	355	73	1.00	1.00	
Yes	31	13	2.04 [1.02–4.08]	1.56 [0.62–3.91]	0.345

NB 1.00 = reference; the bold indicates statistically significant.

## Data Availability

All data used to support the findings of this study are included within the article, and the raw data are available from the corresponding author upon request.

## Abbreviations

ACSH: Ayder comprehensive specialized hospital
AOR: Adjusted odds ratio
BMI: Body mass index
CI: Confidence interval
DM: Diabetes mellitus
CKD: Chronic kidney disease
COR: Crude odds ratio
SCI: Spinal cord injury
UTI: Urinary tract infection.
